# Aortic and arch branch vessel cannulation in acute type A aortic dissection repair

**DOI:** 10.1016/j.xjtc.2022.01.004

**Published:** 2022-01-26

**Authors:** Elizabeth L. Norton, Karen M. Kim, Shinichi Fukuhara, Aroma Naeem, Xiaoting Wu, Himanshu J. Patel, G. Michael Deeb, Bo Yang

**Affiliations:** a Creighton University School of Medicine, Omaha, Neb; b Department of Cardiac Surgery, Michigan Medicine, Ann Arbor, Mich

**Keywords:** acute type A aortic dissection, aorta, cannulation, ABV, arch branch vessel, ACP, antegrade cerebral perfusion, ATAAD, acute type A aortic dissection, CPB, cardiopulmonary bypass, HCA, hypothermic circulatory arrest, LCC, left common carotid, RCP, retrograde cerebral perfusion, RScA, right subclavian artery, RCC, right common carotid, TEE, transesophageal echocardiogram

## Abstract

**Objective:**

To evaluate central aortic cannulation and arch branch vessel (ABV) cannulation in acute type A aortic dissection repair.

**Methods:**

From 2015 to April 2020, 298 patients underwent open repair of an acute type A aortic dissection. Patients undergoing femoral cannulation for cardiopulmonary bypass (n = 34) were excluded. Patients were then divided based on initial cannulation for cardiopulmonary bypass into central aortic cannulation (n = 72) and ABV cannulation (n = 192) groups. ABV sites included cannulation of the axillary, innominate, right/left common carotid, and intrathoracic right subclavian arteries.

**Results:**

The aortic cannulation group was younger (59 vs 62 years; *P* = .02), more likely to be men (76% vs 60%; *P* = .02), and had more peripheral vascular disease (60% vs 37%; *P* = .0009). ABV dissection was similar between central and ABV cannulation groups (53% vs 60%; *P* = .51). The aortic cannulation group underwent less aggressive arch replacement, had shorter cardiopulmonary bypass times (200 vs 222 minutes; *P* = .01), less utilization of antegrade cerebral perfusion (93% vs 98%; *P* = .04), and received less blood transfusion (0 vs 1 U; *P* = .001). Postoperative outcomes were similar between aortic and ABV cannulation groups, including stroke (5.6% vs 5.2%; *P* = 1.0) and operative mortality (4.2% vs 6.3%; *P* = .77). In addition, postoperative strokes were similar in location (right-brain, left-brain, or bilateral), etiology (embolic vs hemorrhagic), and presence of permanent deficits. Aortic cannulation was not a risk factor for postoperative stroke (odds ratio, 0.94; *P* = .91) or operative mortality (odds ratio, 0.70; *P* = .64). Short-term survival was similar between central and ABV cannulation groups.

**Conclusions:**

Both aortic and ABV cannulation were safe and effective cannulation strategies in acute type A aortic dissection repair.

## Graphical abstract

Aortic and arch branch vessel cannulation had similar perioperative and short-term outcomes; therefore, both are safe and effective cannulation strategies in acute type A aortic dissection repair. Each strategy has advantages and disadvantages to consider when determining optimal cannulation strategy for each individual patient and scenario.

**Figure:**
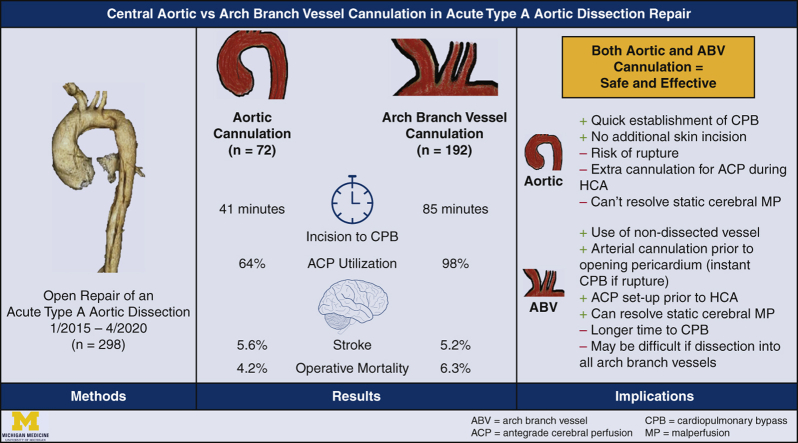

**Figure:**
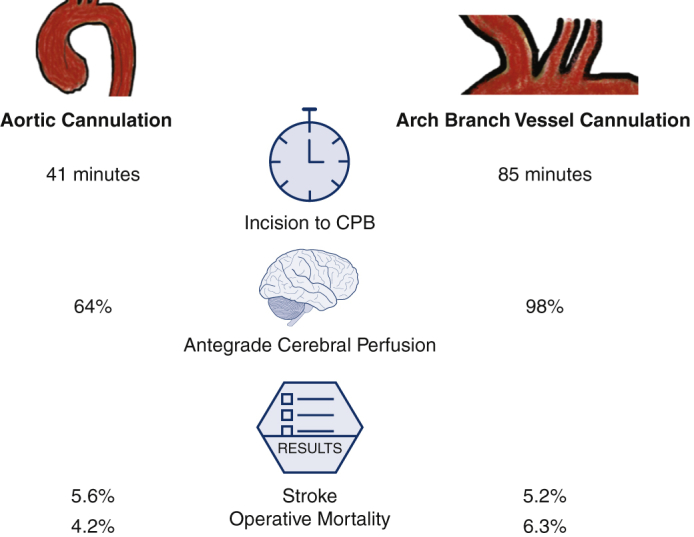
Aortic and arch branch vessel cannulation had similar short-term outcomes.

**Central Message**

Aortic cannulation provides quicker institution of cardiopulmonary bypass with similar outcomes when compared with arch branch vessel cannulation; both cannulation strategies are safe and effective.

**Perspective**

Both central aortic cannulation and arch branch vessel cannulation in acute type A aortic dissection repair are safe and effective strategies. Surgeons should tailor cannulation strategy to each patient's condition and his or her own skill set.

The arterial cannulation strategy in acute type A aortic dissection repair (ATAAD) remains an evolving entity. Traditionally, surgeons started with femoral cannulation and deep hypothermia circulatory arrest (HCA) and retrograde cerebral perfusion (RCP). Gradually, the strategy has evolved to right axillary cannulation and antegrade cerebral perfusion (ACP) and moderate HCA. Direct cannulation of the dissected ascending aorta or aortic arch seemed dangerous. However, aortic cannulation, described in the early 2000s,^1^ has become more and more popular as of late. The results remain inconsistent. Some studies report aortic cannulation as comparable to peripheral cannulation (right axillary artery cannulation or femoral cannulation),^2^^,^^3^ whereas others report worse short- and midterm survival.^4^

At the University of Michigan, in the past 10 years, we have used arch branch vessel (ABV) cannulation, including innominate artery, intrathoracic right subclavian artery (RScA),^5^ right axillary artery, right common carotid (RCC), and left common carotid (LCC) arteries, and direct aortic cannulation for most ATAAD repairs. In this study, we compared the perioperative and short-term outcomes of aortic cannulation and ABV cannulation to determine an optimal cannulation strategy for ATAAD repair. We hypothesized both cannulation strategies would be equally safe and effective.

## Methods

This study was approved by the Institutional Review Board at the University of Michigan, Michigan Medicine (Ann Arbor, Mich) (No. HUM00119716), a waiver of consent was obtained, and was in compliance with Health Insurance Portability and Accountability Act regulations.

### Study Population

Between January 2015 and April 2020, 298 patients underwent open repair of an ATAAD, in which 24% (n = 72) utilized central aortic cannulation and 64% (n = 192) utilized ABV cannulation for institution of cardiopulmonary bypass (CPB). Those with femoral cannulation (11%; n = 34) were reported in the supplemental material (Table E1) to focus on outcomes of aortic and ABV cannulation, which both share the similarity of antegrade blood flow from the aortic arch to distal aorta and lower body during CPB. Aortic cannulation was performed using the Seldinger technique under transesophageal echocardiogram (TEE) guidance, which confirmed location in the true lumen. ABV cannulation included cannulation of the axillary (n = 119), innominate (n = 22), RCC (n = 4), and intrathoracic RScA (n = 47) indirectly with a Dacron graft sewn to the artery and subsequent connecting the Dacron graft to CPB.

Investigators leveraged the Society of Thoracic Surgeons data elements from the University of Michigan Cardiac Surgery Data Warehouse to identify the cohort and determine preoperative, operative, and postoperative characteristics. Electronic medical records were reviewed to supplement data collection. Investigators utilized the National Death Index database through June 30, 2020,^6^ statewide ADT system, medical record review, and telephone call survey (including letters and telephone calls, January 2018) to obtain short-term survival. Loss of follow-up was treated as censors during the time to events analysis. Short-term follow-up was 100% complete until June 30, 2020.

### Surgical Techniques

#### ABV cannulation

ABV cannulation included cannulation of the innominate, intrathoracic RSc,^5^ right axillary, RCC, and LCC arteries. Since 2014, we have used the RScA via an intrathoracic approach more and more for cannulation to avoid another skin incision on the chest wall for the right axillary artery, manipulation of the innominate artery, and a stump of Dacron graft on the innominate artery, especially when the dissection extended into innominate artery but not into the RScA.^5^ RCC or LCC artery cannulation was used when the common carotid artery was dissected and occluded and patients had dynamic or static obstruction of common carotid artery resulting in the cerebral malperfusion and neurological dysfunction, such as strokes. We cannulated the true lumen of common carotid artery distal to obstruction and placed patients on CPB as soon as possible to perfuse the brain quickly. All of the ABV cannulation was achieved with an 8 mm Gelweave Dacron graft (Terumo Aortic Limited) sewn to the ABVs with 5–0 Prolene after heparinization and was connected to the arterial perfusion line with a ¼- to 3/8-inch connector after deairing.

#### Aortic cannulation

The true lumen of the ascending aorta or aortic arch was identified on the computed tomography aortogram and by TEE. A 3–0 or 4–0 Prolene pursestring was placed on the aorta. The true lumen of the aorta was accessed with an 18-gague needle and a guide wire was inserted into the true lumen of the aorta and confirmed by TEE. An EOPA cannula (Medtronic) was then inserted into the true lumen of the aorta and again confirmed by TEE. Sometimes, the true lumen of the aorta can be accessed directly through the nondissected portion of the aorta and other times the true lumen can be accessed through the false lumen and the dissection flap. The operative strategy in patients undergoing ATAAD has been previously described.^7^, ^8^, ^9^

### Statistical Analysis

Initial analysis provided descriptive information on the demographic, clinical, and surgical characteristics. Continuous variables were summarized by median (25%, 75%) and categorical variables were reported as n (%) in frequency tables. Univariate comparisons between aortic and ABV cannulation groups were performed using χ^2^ tests or Fisher exact tests for categorical data and Wilcoxon rank sum tests for continuous data. Univariate analysis was used to assess risk factors for postoperative stroke and operative mortality. Crude survival curves since operation were estimated using the nonparametric Kaplan–Meier method. Log-rank test was used to compare the survival between groups. The variables for univariate analysis were selected based on clinical judgment. All statistical calculations used SAS version 9.4 (SAS Institute Inc) and were considered significant at *P* < .05.

## Results

### Demographics/Preoperative Data

The central aortic cannulation group was younger (59 vs 62 years; *P* = .02), more likely to be men (76% vs 60%; *P* = .02), had higher body mass index (31 vs 28; *P* = .009), and more peripheral vascular disease (60% vs 37%; *P* = .001), and less cardiac tamponade (5.6% vs 19%; *P* = .006) compared with the ABV cannulation group. Other preoperative characteristics, including hypertension, connective tissue disorder, previous cardiac surgery, and malperfusion syndrome were similar between groups (Table 1). The proportion of DeBakey type II dissections was almost twice as high in the aortic cannulation group compared with the ABV group (17% vs 9.9%; *P* = .13). Dissection into the innominate (50% vs 57%; *P* = .30) and LCC (24% vs 32%; *P* = .19) arteries was also similar between aortic and ABV cannulation groups.

**Table 1:** Demographic characteristics and preoperative outcomes

Characteristic or outcome	Total (n = 264)	Aortic (n = 72)	ABV (n = 192)	*P* value∗
Patient age (y)	**61 (51, 70)**	**59 (49, 68)**	**62 (53, 72)**	**.02**
Male sex	**171 (65)**	**55 (76)**	**116 (60)**	**.02**
BMI	**29 (25, 34)**	**31 (26, 36)**	**28 (24, 33)**	**.009**
Preexisting comorbidities				
Hypertension	220 (83)	60 (83)	160 (83)	1.0
Diabetes	25 (9.5)	7 (9.7)	18 (9.4)	.93
Smoking status				.30
Never	89 (34)	27 (38)	62 (32)	.44
Former	81 (31)	17 (24)	64 (34)	.12
Current	93 (35)	28 (39)	65 (34)	.46
CAD	43 (17)	13 (18)	30 (16)	.71
COPD	39 (15)	10 (14)	29 (15)	.80
History of MI	19 (7.2)	7 (9.7)	12 (6.3)	.33
History of renal failure	13 (4.9)	4 (5.6)	9 (4.7)	.76
History of CVA	16 (6.1)	4 (5.6)	12 (6.3)	1.0
PVD	**114 (43)**	**43 (60)**	**71 (37)**	**.0009**
Connective tissue disorder	5 (1.9)	0 (0)	5 (2.6)	.33
Bicuspid aortic valve	20 (7.7)	6 (8.3)	14 (7.4)	.78
Previous cardiac surgery	20 (7.6)	5 (6.9)	15 (7.8)	.81
Preoperative AI				.25
None	62 (24)	18 (25)	44 (23)	.75
Trace	27 (10)	7 (9.7)	20 (11)	.84
Mild	75 (29)	27 (38)	48 (25)	.05
Moderate	53 (20)	10 (14)	43 (23)	.12
Severe	45 (17)	10 (14)	35 (18)	.39
Ejection fraction	58 (55, 65)	58 (55, 65)	57 (55, 64)	.09
Acute MI	8 (3.0)	4 (5.6)	4 (2.1)	.22
Acute stroke	32 (12)	5 (6.9)	27 (14)	.11
Acute renal insufficiency	20 (7.6)	7 (9.7)	13 (6.8)	.42
Acute paralysis	6 (2.3)	2 (2.8)	4 (2.1)	.67
Cardiogenic shock	22 (8.3)	3 (4.2)	19 (9.9)	.13
Tamponade	**41 (16)**	**4 (5.6)**	**37 (19)**	**.006**
CPR	5 (1.9)	1 (1.4)	4 (2.1)	1.0
Preoperative creatinine	1.0 (0.8, 1.3)	1.0 (0.8, 1.4)	1.0 (0.8, 1.3)	.49
Malperfusion syndrome	76 (29)	20 (28)	56 (29)	.82
Coronary	9 (3.4)	4 (5.6)	5 (2.6)	.26
Cerebral	31 (12)	5 (6.9)	26 (14)	.14
Spinal cord	2 (0.8)	1 (1.4)	1 (0.5)	.47
Celiac	7 (2.7)	4 (5.6)	3 (1.6)	.09
Mesenteric	24 (9.1)	9 (13)	15 (7.8)	.24
Renal	23 (8.7)	7 (9.7)	16 (8.3)	.72
Lower extremity	23 (8.7)	8 (11)	15 (7.8)	.40
Delayed operation	30 (11)	9 (13)	21 (11)	.72
DeBakey class				.13
I	233 (88)	60 (83)	173 (90)	
II	31 (12)	12 (17)	19 (9.9)	
ABV dissection	152 (58)	38 (53)	114 (60)	.51
Innominate	145 (55)	36 (50)	109 (57)	.30
Left common carotid	78 (30)	17 (24)	61 (32)	.19

Values are presented as median (25%, 75%) for continuous data and n (%) for categorical data. Bold font indicates statistical significance. *ABV*, Arch branch vessel; *BMI*, body mass index; *CAD*, coronary artery disease; *COPD*, chronic obstructive pulmonary disease; *MI*, myocardial infarction; *CVA*, cerebrovascular accident; *PVD*, peripheral vascular disease; *AI*, aortic insufficiency; *CPR*, cardiopulmonary resuscitation.

∗ *P* value indicates the difference between aortic cannulation and ABV cannulation groups.

### Operative Data

There was no intraoperative aortic rupture due to aortic cannulation. The surgical incision to CPB time was significantly shorter in the aortic cannulation group (41 vs 85 minutes; *P* < .0001). Although aortic root procedures were similar between groups, the aortic cannulation group underwent significantly less extensive arch procedures, specifically 6.9% of patients in the aortic cannulation group underwent no aortic arch procedure compared with 1.6% of patients in the ABV cannulation group (*P* = .04) and the aortic cannulation group underwent less zone 1 arch replacement (1.4% vs 15%; *P* = .002). The aortic cannulation group had shorter CPB times (200 vs 222 minutes; *P* = .01) and less utilization of hypothermic circulatory arrest (93% vs 98%; *P* = .04). The HCA (28 vs 28 minutes) and crossclamp times were very similar between the 2 groups. Twenty-nine percent of cases in the aortic cannulation group had only RCP and 95% cases in the ABV group had only ACP. In the ABV group, there was no direct carotid cannulation through the open aortic arch. In the aortic cannulation group, all of the ACP was achieved through direct innominate or carotid artery cannulation (total 46 patients, including 33 patients with ACP and 13 patients with RCP + ACP). The lowest temperature was similar in both groups (23 vs 22 °C). The aortic cannulation group required less intraoperative transfusion of packed red blood cells (0 vs 1 U; *P* = .001) (Table 2).

**Table 2:** Intraoperative data

	Total (n = 264)	Aortic (n = 72)	ABV (n = 192)	*P* value∗
Aortic root procedure				.62
None	20 (7.6)	3 (4.2)	17 (8.9)	.20
AVR only	9 (3.4)	1 (1.4)	8 (4.2)	.27
Root replacement	79 (30)	24 (33)	55 (29)	.46
Root repair	156 (59)	44 (61)	112 (58)	.68
Arch replacement				**.004**
None	**8 (3.0)**	**5 (6.9)**	**3 (1.6)**	**.04**
Hemiarch	162 (61)	50 (69)	112 (58)	.10
Zone 1 Arch	**29 (11)**	**1 (1.4)**	**28 (15)**	**.002**
Zone 2 Arch	47 (18)	10 (14)	37 (19)	.35
Zone 3 Arch	18 (6.8)	6 (8.3)	12 (6.3)	.59
Frozen elephant trunk	69 (26)	18 (25)	51 (26)	.80
Concomitant procedures				.42
CABG	14 (5.3)	4 (5.6)	10 (5.2)	1.0
Mitral valve	6 (2.3)	3 (4.2)	3 (1.6)	.35
Tricuspid valve	2 (0.8)	0 (0)	2 (1.0)	1.0
Surgical incision to CPB (min)	**75 (60, 95)**	**41 (33, 61)**	**85 (72, 100)**	**<.0001**
CPB time (min)	**217 (179, 270)**	**200 (163, 251)**	**222 (184, 279)**	**.01**
Crossclamp time (min)	148 (110, 200)	144 (109, 181)	150 (113, 204)	.34
HCA	**256 (97)**	**67 (93)**	**189 (98)**	**.04**
HCA time (min)	28 (21, 41)	28 (18, 43)	28 (22, 40)	.41
Cerebral perfusion				**<.001**
Antegrade	**216 (82)**	**33 (46)**	**183 (95)**	**<.0001**
Retrograde	**21 (8.0)**	**21 (29)**	**0 (0)**	**<.0001**
Both antegrade and retrograde	**19 (7.2)**	**13 (18)**	**6 (3.1)**	**<.0001**
Lowest temperature (°C)	22 (18, 25)	23 (19, 25)	22 (18, 25)	.53
Blood transfusion (PRBC units)	**1.0 (0.0, 3.0)**	**0.0 (0.0, 1.0)**	**1.0 (0.0, 3.0)**	**.001**

Values are presented as median (25%, 75%) for continuous data and n (%) for categorical data. Bold font indicates statistical significance. *ABV*, Arch branch vessel; *AVR*, aortic valve replacement; *CABG*, coronary artery bypass graft; *CPB*, cardiopulmonary bypass; *HCA*, hypothermic circulatory arrest; *PRBC*, packed red blood cells.

∗ *P* value indicates the difference between aortic cannulation and ABV cannulation groups.

### Postoperative Outcomes

Postoperative outcomes were similar between aortic and ABV cannulation groups, including stroke (5.6% vs 5.2%; *P* = 1.0), intraoperative mortality (0% vs 0%; *P* = 1.0), and operative mortality (4.2% vs 6.3%; *P* = .77) (Table 3). Postoperative strokes were similar between aortic and ABV cannulation groups in location (left-brain: 0% vs 0%, right-brain: 2.8% vs 2.1%, and bilateral: 2.8% vs 3.1%; *P* = 1.0), etiology (embolic: 4.2% vs 4.7% and hemorrhagic: 1.4% vs 0.5%; *P* = .51), and influence (permanent deficit[s]: 1.4% vs 2.6%; *P* = .58) (Table 4). Aortic cannulation was not a risk factor for postoperative stroke (odds ratio [OR], 0.88; 95% CI, 0.28-2.75; *P* = .82) or operative mortality (OR, 1.38; 95% CI, 0.40-4.68; *P* = .61) by univariate analysis (Table 5). Preoperative acute stroke (OR, 5.77; 95% CI, 1.95-17.1; *P* = .002), preoperative acute renal failure (OR, 5.54; 95% CI, 1.63-18.8; *P* = .006), and preoperative acute paralysis (OR, 10.1; 95% CI, 1.74-58.6; *P* = .01) were risk factors for operative mortality by univariate analysis.

**Table 3:** Postoperative data

	Total (n = 264)	Aortic (n = 72)	ABV (n = 192)	*P* value∗
Reoperation for bleeding	8 (3.0)	2 (2.8)	6 (3.1)	1.0
Tamponade	1 (0.4)	0 (0)	1 (0.5)	1.0
Deep sternal wound infection	1 (0.4)	0 (0)	1 (0.5)	1.0
Sepsis	6 (2.3)	3 (4.2)	3 (1.6)	.35
Postoperative MI	2 (0.8)	1 (1.4)	1 (0.5)	.47
Atrial fibrillation	93 (35)	27 (38)	66 (34)	.64
Cerebrovascular accident	14 (5.3)	4 (5.6)	10 (5.2)	1.0
TIA	1 (0.4)	0 (0)	1 (0.5)	1.0
New-onset paraplegia	1 (0.4)	0 (0)	1 (0.5)	1.0
Acute renal insufficiency	41 (16)	12 (17)	29 (15)	.75
Requiring dialysis	22 (8.3)	8 (11)	14 (7.3)	.32
Permanent	11 (4.2)	6 (8.3)	5 (2.6)	.18
Gastrointestinal complications	27 (10)	6 (8.3)	21 (11)	.53
Pneumonia	41 (16)	8 (11)	33 (17)	.22
Prolonged ventilation >24 h	128 (48)	31 (43)	97 (51)	.28
Hours intubated	32 (18, 81)	27 (16, 74)	34 (19, 81)	.25
Reintubation	21 (8.0)	5 (6.9)	16 (8.3)	.71
Tracheostomy	6 (2.3)	3 (4.2)	3 (1.6)	.35
Postoperative LOS (d)	11 (7.0, 16)	10 (7.0, 18)	11 (7.0, 16)	.95
Total LOS (d)	11 (7.0, 18)	11 (7.0, 18)	12 (7.5, 18)	.78
Intraoperative mortality	0 (0)	0 (0)	0 (0)	1.0
In-hospital mortality	13 (4.9)	3 (4.2)	10 (5.2)	1.0
30-d mortality	13 (4.9)	2 (2.8)	11 (5.7)	.52
Operative mortality†	15 (5.7)	3 (4.2)	12 (6.3)	.77

Values are presented as median (25%, 75%) for continuous data and n (%) for categorical data. *ABV*, Arch branch vessel; *MI*, myocardial infarction; *TIA*, transient ischemic attack; *LOS*, length of stay.

∗ *P* value indicates the difference between aortic cannulation and ABV cannulation groups.

† Operative mortality includes 30-day mortality and/or in-hospital mortality.

**Table 4:** Details of postoperative stroke amongst groups

	Aortic (n = 72)	ABV (n = 192)	*P* value∗
Stroke	4 (5.6)	10 (5.2)	1.0
Location			
Left-brain	0 (0)	0 (0)	1.0
Right-brain	2 (2.8)	4 (2.1)	1.0
Both sides	2 (2.8)	6 (3.1)	1.0
Etiology			
Embolic	3 (4.2)	9 (4.7)	.51
Hemorrhagic	1 (1.4)	1 (0.5)	.51
Permanent†	1 (1.4)	5 (2.6)	.58

Univariate comparisons were performed using χ^2^ tests for categorical data. *ABV*, Arch branch vessel.

∗ *P* value indicates the difference between aortic cannulation and ABV cannulation groups.

† Permanent stroke was defined as stroke not fully recovered at postoperative visit or before in-hospital death.

**Table 5:** Risk factors for postoperative stroke and operative mortality by univariate analysis

	Odds ratio (95% CI)	*P* value
Postoperative stroke		
Aortic cannulation	0.88 (0.28-2.75)	.82
ABV dissection	0.72 (0.25-2.05)	.63
Acute stroke	0.23 (0.01-4.16)	.32
Age	0.98 (0.94-1.02)	.22
Male sex	0.77 (0.25-2.42)	.66
CAD	1.54 (0.44-5.39)	.50
Cardiogenic shock	2.25 (0.52-9.67)	.28
Operative mortality		
Aortic cannulation	1.38 (0.40-4.68)	.61
Age	1.03 (0.99-1.07)	.16
Male sex	0.70 (0.23-2.16)	.54
Acute stroke	**5.77 (1.95-17.1)**	**.002**
CAD	1.41 (0.41-4.88)	.59
COPD	1.64 (0.47-5.71)	.44
Acute renal failure	**5.54 (1.63-18.8)**	**.006**
Cardiogenic shock	3.31 (0.91-12.1)	.07
Acute paralysis	**10.1 (1.74-58.6)**	**.01**

Bold font indicates statistical significance.

*ABV*, Arch branch vessel; *CAD*, coronary artery disease; *COPD*, chronic obstructive pulmonary disease.

### Short-Term Outcomes

**Figure 1:**
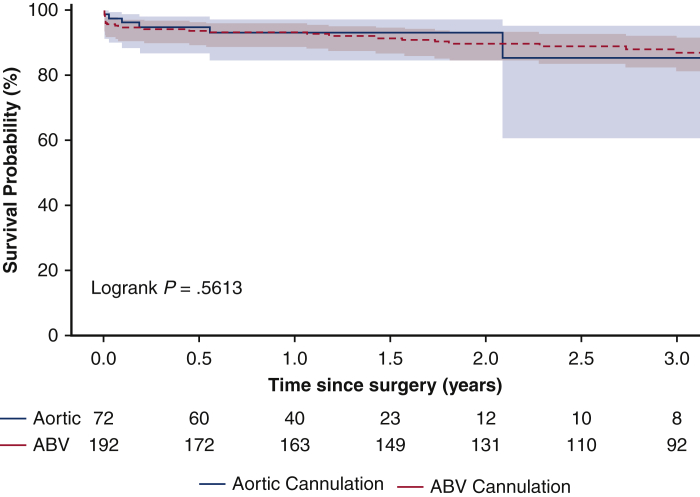
Kaplan-Meier survival analysis of patients undergoing open acute type A aortic dissection repair with either central aortic cannulation or arch branch vessel (*ABV*) cannulation for institution of cardiopulmonary bypass. One-year survival since operation was statistically similar between central aortic (93%, 95% CI, 84%-97%) and ABV cannulation groups (92%, 95% CI, 87%-95%) (*P* = .71).

The mean follow-up time was 2.4 ± 1.6 years. The 1-year survival was similar between central aortic and ABV cannulation groups (93%, 95% CI, 84%, 97% vs 92%, 95% CI, 87%, 95%) (Figure 1).

## Discussion

In this study, we found that aortic cannulation enabled quicker CPB institution. Despite the aortic cannulation group having simpler aortic arch procedures, the HCA time was very similar between the 2 groups. The perioperative and short-term outcomes were similar between the 2 cannulation strategies. Aortic cannulation was not a significant risk factor compared with ABV cannulation for operative mortality.

Arterial cannulation for CPB is very important in the surgical repair of ATAAD. A good cannulation strategy should completely perfuse all tissues and organs in a patient when CPB is initiated; not cause malperfusion but resolve malperfusion if patients have any caused by ATAAD, and not cause aortic rupture. The arterial cannulation strategy is also influenced by the anticipated strategy of cerebral protection, such as using ACP or RCP and deep HCA at 18 °C or moderate HCA at 28 °C, or an even higher nasopharyngeal temperature. Surgeons using ABV cannulation usually use unilateral ACP and could add another cannula into the LCC if bilateral ACP is needed, such as in our study, 98% of cases in the ABV group utilized ACP. With aortic cannulation, the ACP is not set up before HCA. Frequently, surgeons use deep HCA with RCP only (29% cases in the aortic cannulation group in this study) (Table 2). If surgeons want to perform ACP, they have to cannulate the innominate artery or the common carotid artery individually with direct insertion of the cannula into the true lumen of ABVs as an additional step. That was likely why the HCA time was similar in both groups despite the aortic cannulation group had simpler arch reconstruction (more hemiarch replacement and less zone 1 arch replacement).

Arterial cannulation strategy has evolved over time, with increasing use of ABV and aortic cannulation, and decreasing use of femoral cannulation.^10^ Cannulation strategy depends on a variety of clinical factors, including extent of dissection into branch vessels, presence of malperfusion, vessel calcification, and hemodynamic stability, as well as surgeon skill sets. There are advantages and disadvantages of both ABV cannulation and aortic cannulation in ATAAD repair (Table 6).

**Table 6:** Comparison aortic and arch branch vessel (ABV) cannulation

	Aortic cannulation	ABV cannulation
Advantages	Fast implementation of CPB to resolve cardiac tamponade or dynamic malperfusion No additional skin incision	Cannulation of nondissected vessel Can be set up before opening the pericardium, allowing immediate CPB if aorta ruptures ACP already set up to reduce the HCA time Can resolve static cerebral malperfusion immediately
Disadvantages	Could cause aortic rupture Have to open pericardium for cannulation, no arterial line ready for CPB if aorta ruptures ACP requires an extra step for individual ABV cannulation Cannot resolve static cerebral malperfusion	Longer time from incision to CPB Additional skin incision, injury to the brachial plexus, or insufficient caliber for full CPB, if the axillary artery is used. Those can be avoided if the innominate artery or intrathoracic right subclavian artery is used May not be available if dissection extends into all ABVs

*CPB*, Cardiopulmonary bypass; *ACP*, antegrade cerebral perfusion; *HCA*, hypothermic circulatory arrest.

The advantages of using ABV are:

• The arterial cannulation is done in a nondissected, normal branch vessel of the arch with an 8 mm Dacron graft sewn to it. This technique is very safe and effective to deliver the blood flow into the true lumen during CPB.
• The ABV arterial cannulation can be set up before opening the pericardium. Therefore, surgeons can put a patient on CPB immediately in case the aorta ruptures when opening the pericardium.
• Once the ABV is cannulated, ACP is already set up through the right axillary artery, RScA, innominate artery, or RCC artery by clamping the proximal innominate artery, or through LCC artery by clamping the LCC artery proximal to the cannulation site when the LCC artery is used for cannulation in patients with LCC artery static malperfusion or when the innominate artery and its branches are not suitable for cannulation. There is no need for direct manipulation of the ABVs to insert the cannula directly into the innominate or LCC artery. Minimizing the manipulation of the ABVs could decrease the risk of stroke as shown in our previous study.^11^ When complex aortic arch reconstruction is needed, although rare, it is also very easy to convert to bilateral ACP by inserting a cannula directly into the carotid artery that is not perfused. Alternatively, brief deep HCA with a debranching graft sewn to the innominate artery followed by ACP can be utilized, as describe by other centers.^12^^,^^13^
• Once ACP is set up, we frequently use moderate HCA at a nasopharyngeal temperature of 28 °C to minimize CPB time and inflammation from CPB.
• When the aortic dissection extends into the common carotid arteries and the false lumen is thrombosed resulting in static cerebral malperfusion, the only way to perfuse the brain quickly is to cannulate the common carotid artery distal to the occluded portion of the common carotid artery and put patients on CPB to salvage the brain and replace the dissected common carotid artery after aortic arch reconstruction^9^^,^^14^ assuming the internal carotid artery is not dissection and occluded.^15^

The disadvantages of using ABVs for arterial cannulation are:

• It takes longer from skin incision to the initiation of CPB (Table 2).
• In the case of axillary artery cannulation, it requires an additional skin incision and there is a risk of injury to the brachial plexus. But this can be avoided by using the intrathoracic RScA^5^ or innominate artery.
• In the case of RScA cannulation, there is risk of injury to the right recurrent laryngeal nerve, but we have not seen this complication.^5^
• If the aortic dissection extends into the right axillary artery, RCC artery, and LCC artery (which we have not seen), then there may not be an ABV available for surgeons to use for cannulation. However, we have cannulated a dissected right axillary artery by sewing an 8 mm Dacron graft to the true lumen of right axillary artery for CPB.
• Sometimes the right axillary artery could be small and limit the blood flow for CPB, especially in small women; in this situation, an additional aortic cannulation could solve the problem.

The advantages of aortic cannulation are:

• Fast implementation of CPB. There is no need to extensively dissect out ABVs unless surgeons decide to replace those ABVs. Once surgeons are familiar with it, it can be done quickly; therefore, timing could be operator dependent. This strategy could be useful when patients are quickly decompensating, unstable due to cardiac tamponade, or rupture and need CPB immediately.
• No additional skin incision.
• It achieves true lumen perfusion and could resolve the dynamic malperfusion quickly. At our center, we resolved malperfusion endovascularly if patients develop malperfusion syndrome and organ failure before open aortic repair^16^; therefore, we did not see this advantage in our patients.

The disadvantages are:

• Potentially causing aortic rupture during cannulation as reported by others.^1^^,^^17^ We have not had such complication in our series nor did other recent studies.^3^^,^^18^
• Surgeons have to open the pericardium to cannulate the aorta. The dissected ascending aorta could rupture when the pericardium is being opened and there is no arterial line ready to initiate CPB.
• The ACP is not set up before the HCA. Surgeons either have to use deep HCA at 18 to 20 °C with or without RCP or insert a cannula into the individual ABVs for ACP. Direct cannulation of ABVs could increase HCA time and cause embolic strokes by dislodging thrombus in the false lumen of the dissected innominate or carotid artery. If the cannula in the innominate artery is too deep, it could go down to the RScA, resulting in lack of perfusion in the RCC artery. Another good strategy described by other groups is to place a DLP needle (Medtronic) in a nondissected innominate artery for ACP with direct aortic cannulation.^19^
• Aortic cannulation cannot resolve static malperfusion of the common carotid artery due to thrombosed false lumen.

There are those who advocate for using aortic cannulation over other cannulation strategies in patients with cardiac tamponade or cerebral malperfusion.^14^^,^^20^ However, our cannulation strategies (aortic vs ABV cannulation) are not influenced by those conditions. At our institution, 2 surgeons used aortic cannulation as their go-to strategy, including patients with or without cardiac tamponade or cerebral malperfusion. One surgeon uses ABV cannulation as the first choice in all patients undergoing ATAAD, including patients with cardiac tamponade or cerebral malperfusion. In patients with cardiac tamponade and who are unstable, we opened the pericardium and resolved the tamponade. After the patients became more stable, we then performed ABV cannulation for the benefit discussed above (Table 6). Most of the time we cannulated the ABV before opening the pericardium, even in patients with cardiac tamponade in case the aorta ruptures upon open the pericardium. In this study, the ABV cannulation group had significantly more cardiac tamponade than the aortic cannulation group (19% vs 5.6%; *P* = .006) (Table 2), but the operative mortality was similar (6.3% vs 4.2%; *P* = .77) (Table 3). In patients with proximal aortic rupture who need emergent cannulation, aortic cannulation can initiate CPB quickly and the aorta can be crossclamped distal to the rupture. Some surgeons would choose femoral artery/vein cannulation in this situation with chest compressions to perfuse the brain continuously without interruption.

In patients with cerebral malperfusion, we cannulated the occluded carotid artery distal to the occlusion through a separate incision and initiated CPB immediately to guarantee cerebral perfusion. In cases of static malperfusion of a thrombosed carotid artery, aortic cannulation may not resolve the carotid malperfusion. In this study, we also had double the amount of cerebral malperfusion in the ABV cannulation group compared with the aortic cannulation group (14% vs 7%) (Table 2), but postoperative stroke rate was similar (5.2 vs 5.6%) (Table 3).

Both aortic and ABV cannulation achieve antegrade blood flow during CPB. However, aortic dissection is a dynamic phenomenon. New dissection and new malperfusion can develop during CPB. It is very important to monitor the perfusion of both the upper and lower body simultaneously. At our institution, we routinely place a right radial and femoral arterial line to monitor upper and lower body blood pressure. If the lower body is malperfused after CPB initiation, we cannulate the femoral artery where the low arterial blood pressure is detected by sewing a Dacron graft to the artery to perfuse the lower body.

**Figure 2:**
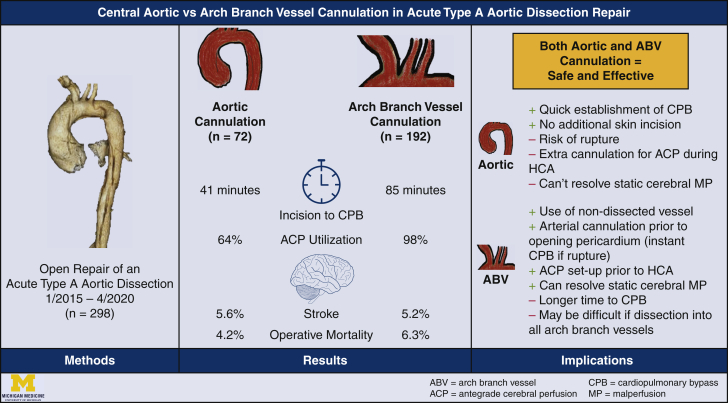
Aortic cannulation and arch branch vessel (*ABV*) cannulation had similar perioperative and short-term outcomes; therefore, both are safe and effective cannulation strategies in acute type A aortic dissection repair. Each strategy has advantages and disadvantages to consider when determining optimal cannulation strategy for each individual patient and scenario. *CPB*, Cardiopulmonary bypass; *ACP*, antegrade cerebral perfusion; *HCA*, hypothermic circulatory arrest; *MP*, malperfusion.

In summary, there was no difference in the perioperative and short-term outcomes between the 2 cannulation strategies, including postoperative stroke, new-onset dialysis, operative mortality, and short-term mortality (Table 3). As discussed above, both aortic cannulation and ABV cannulation have their advantages and disadvantages. Surgeons should be familiar with various cannulation techniques and strategies and tailor the cannulation strategy to each individual patient (Figure 2).

This study is limited by its retrospective nature and the relatively small sample size of the aortic cannulation group. The small sample size could cause type II error due to inadequate statistical power. It was not a randomized trial and the cannulation strategy was based on surgeon's preference. We presented the results in a descriptive way (Video 1).

**Video 1:**
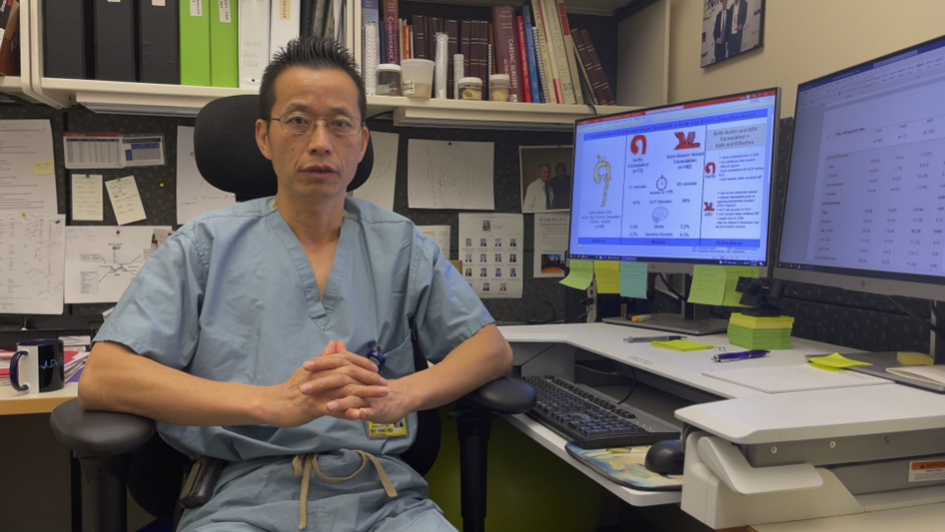
Discussion of the outcomes of aortic cannulation and aortic arch branch vessel cannulation. Video available at: https://www.jtcvs.org/article/S2666-2507(22)00030-X/fulltext.

## Conclusions

Perioperative and short-term outcomes were similar between the aortic cannulation and ABV cannulation groups. Both aortic and ABV cannulation were safe and effective cannulation strategies in ATAAD repair. Surgeons should tailor the cannulation strategy to each individual patient and their own skill sets.

### Conflict of Interest Statement

The authors reported no conflicts of interest.

The *Journal* policy requires editors and reviewers to disclose conflicts of interest and to decline handling or reviewing manuscripts for which they may have a conflict of interest. The editors and reviewers of this article have no conflicts of interest.

## Figures and Tables

**Table E1:** Postoperative data with femoral cannulation

	Aortic (n = 72)	ABV (n = 192)	Femoral (n = 34)
Reoperation for bleeding	2 (2.8)	6 (3.1)	3 (8.8)
Tamponade	0 (0)	1 (0.5)	1 (2.9)
Deep sternal wound infection	0 (0)	1 (0.5)	0 (0)
Sepsis	3 (4.2)	3 (1.6)	1 (2.9)
Postoperative MI	1 (1.4)	1 (0.5)	0 (0)
Atrial fibrillation	27 (38)	66 (34)	11 (32)
Cerebrovascular accident	4 (5.6)	10 (5.2)	5 (15)
TIA	0 (0)	1 (0.5)	0 (0)
New-onset paraplegia	0 (0)	1 (0.5)	0 (0)
Acute renal insufficiency	12 (17)	29 (15)	8 (24)
Requiring dialysis	8 (11)	14 (7.3)	5 (15)
Permanent	6 (8.3)	5 (2.6)	2 (5.9)
Gastrointestinal complications	6 (8.3)	21 (11)	5 (15)
Pneumonia	8 (11)	33 (17)	5 (15)
Prolonged ventilation (>24 h)	31 (43)	97 (51)	15 (44)
Hours intubated	27 (16, 74)	34 (19, 81)	29 (21, 85)
Reintubation	5 (6.9)	16 (8.3)	2 (5.9)
Tracheostomy	3 (4.2)	3 (1.6)	0 (0)
Postoperative LOS (d)	10 (7.0, 18)	11 (7.0, 16)	8.0 (6.3, 17)
Total LOS (d)	11 (7.0, 18)	12 (7.5, 18)	8.0 (7.0, 17)
Intraoperative mortality	0 (0)	0 (0)	2 (5.9)
In-hospital mortality	3 (4.2)	10 (5.2)	3 (8.8)
30-d mortality	2 (2.8)	11 (5.7)	2 (5.9)
Operative mortality∗	3 (4.2)	12 (6.3)	3 (8.8)

Values are presented as median (25%, 75%) for continuous data and n (%) for categorical data. *ABV*, Arch branch vessel; *MI*, myocardial infarction; *TIA*, transient ischemic attack; *LOS*, length of stay.

∗ Operative mortality includes 30-day mortality and/or in-hospital mortality.

## Supplementary Data

Video 1 Discussion of the outcomes of aortic cannulation and aortic arch branch vessel cannulation. Video available at: https://www.jtcvs.org/article/S2666-2507(22)00030-X/fulltext.

## References

[1] Reece T.B., Tribble C.G., Smith R.L., Singh R.R., Stiles B.M., Peeler B.B. (2017). Central cannulation is safe in acute aortic dissection repair. J Thorac Cardiovasc Surg.

[2] Klotz S., Heuermann K., Hanke T., Petersen M., Sievers H.H. (2015). Outcome with peripheral versus central cannulation in acute type A dissection dagger. Interact Cardiovasc Thorac Surg.

[3] Kreibich M., Chen Z., Rylski B., Bavaria J.E., Brown C.R., Branchetti E. (2019). Outcome after aortic, axillary, or femoral cannulation for acute type A aortic dissection. J Thorac Cardiovasc Surg.

[4] Sabashnikov A., Heinen S., Deppe A.C., Zeriouh M., Weymann A., Slottosch I. (2016). Axillar or aortic cannulation for aortic repair in patients with Stanford A dissection?. Ann Thorac Surg.

[5] Norton E.L., Makkinejad A., Le T., Wu X., Yang B. (2020). Intrathoracic right subclavian artery cannulation in aortic arch surgery. J Thorac Cardiovasc Surg Tech.

[6] Centers for Disease Control and Prevention, National Center for Health Statistics National Death Index. http://www.cdc.gov/nchs/ndi/index.htm.

[7] Yang B., Norton E.L., Hobbs R., Farhat L., Wu X., Hornsby W.E. (2019). Short- and long-term outcomes of aortic root repair and replacement in patients undergoing acute type A aortic dissection repair: 20-year experience. J Thorac Cardiovasc Surg.

[8] Yang B., Norton E.L., Shih T., Farhat L., Wu X., Hornsby W.E. (2019). Late outcomes of strategic arch resection in acute type A aortic dissection. J Thorac Cardiovasc Surg.

[9] Norton E.L., Wu X., Farhat L., Kim K.M., Patel H.J., Deeb G.M. (2019). Dissection of arch branches alone an indication for aggressive arch management in type A dissection?. Ann Thorac Surg.

[10] Zhu Y., Lingala B., Baiocchi M., Tao J.J., Toro Arana V., Khoo J.W. (2020). Type A aortic dissection-experience over 5 decades: JACC historical breakthroughs in perspective. J Am Coll Cardiol.

[11] Norton E.L., Wu X., Kim K.M., Patel H.J., Deeb G.M., Yang B. (2020). Unilateral is comparable to bilateral antegrade cerebral perfusion in acute type A aortic dissection repair. J Thorac Cardiovasc Surg.

[12] Aalaei-Andabili S.H., Martin T., Hess P., Lee T., Arnaoutakis G., Beaver T.M. (2019). Even redo ascending aorta replacement has low mortality in elective setting. J Cardiovasc Surg (Torino).

[13] Falasa M.P., Arnaoutakis G.J., Janelle G.M., Beaver T.M. (2021). Neuromonitoring and neuroprotection advances for aortic arch surgery. J Thorac Cardiovasc Surg Tech.

[14] Trivedi D., Navid F., Balzer J.R., Joshi R., Lacomis J.M., Jovin T.G. (2016). Aggressive aortic arch and carotid replacement strategy for type A aortic dissection improves neurologic outcomes. Ann Thorac Surg.

[15] Fukuhara S., Norton E.L., Chaudhary N., Burris N., Shiomi S., Kim K.M. (2021). Type A aortic dissection with cerebral malperfusion: new insights. Ann Thorac Surg.

[16] Yang B., Rosati C.M., Norton E.L., Kim K.M., Khaja M.S., Dasika N. (2018). Endovascular fenestration/stenting first followed by delayed open aortic repair for acute type A aortic dissection with malperfusion syndrome. Circulation.

[17] Khaladj N., Shrestha M., Peterss S., Strueber M., Karck M., Pichlmaier M. (2008). Ascending aortic cannulation in acute aortic dissection type A: the Hannover experience. Eur J Cardiothorac Surg.

[18] El Beyrouti H., Dohle D.S., Izzat M.B., Brendel L., Pfeiffer P., Vahl C.F. (2020). Direct true lumen cannulation in type A acute aortic dissection: a review of an 11 years' experience. PLoS One.

[19] Payabyab E.C., Hemli J.M., Mattia A., Kremers A., Vatsia S.K., Scheinerman S.J. (2020). The use of innominate artery cannulation for antegrade cerebral perfusion in aortic dissection. J Cardiothorac Surg.

[20] Rylski B., Urbanski P.P., Siepe M., Beyersdorf F., Bachet J., Gleason T.G. (2014). Operative techniques in patients with type A dissection complicated by cerebral malperfusion. Eur J Cardiothorac Surg.

